# Supplementary material for: Cas9-assisted biological containment of a genetically engineered human commensal bacterium and genetic elements
Source: Nat Commun. 2024 Mar 7;15:2096. doi: 10.1038/s41467-024-45893-w (PMC10920895; doi:10.1038/s41467-024-45893-w)
Supplement: Supplementary file 1 — Supplementary Information [file 41467_2024_45893_MOESM1_ESM.pdf]

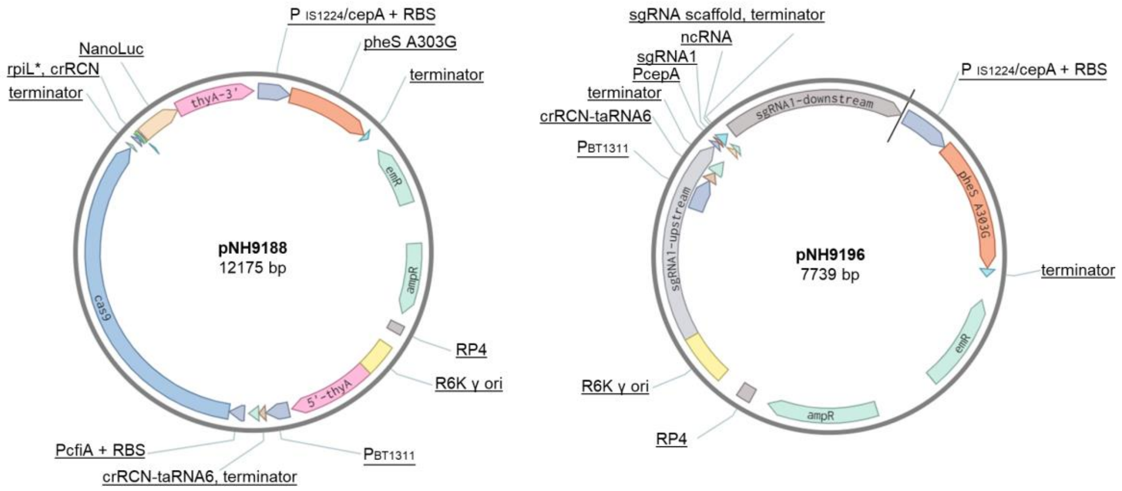


**Supplementary Fig. 1 Genetic maps of representative plasmids used in this study**. Left, pNH9188, used for the first recombination step to integrate the components of the Engineered Riboregulator (ER), i.e., the trans-activating RNA sequence (taRNA6) and the cis-repressive sequence (crRCN), as well as the SpCas9 gene, the NanoLuc gene, and their promoters into the chromosome of *Bacteroides thetaiotaomicron*. Right, pNH9196, used for the second recombination step, to integrate the single-guide RNA sequence (sgRNA1) and its promoter ($P_{cepA}$).

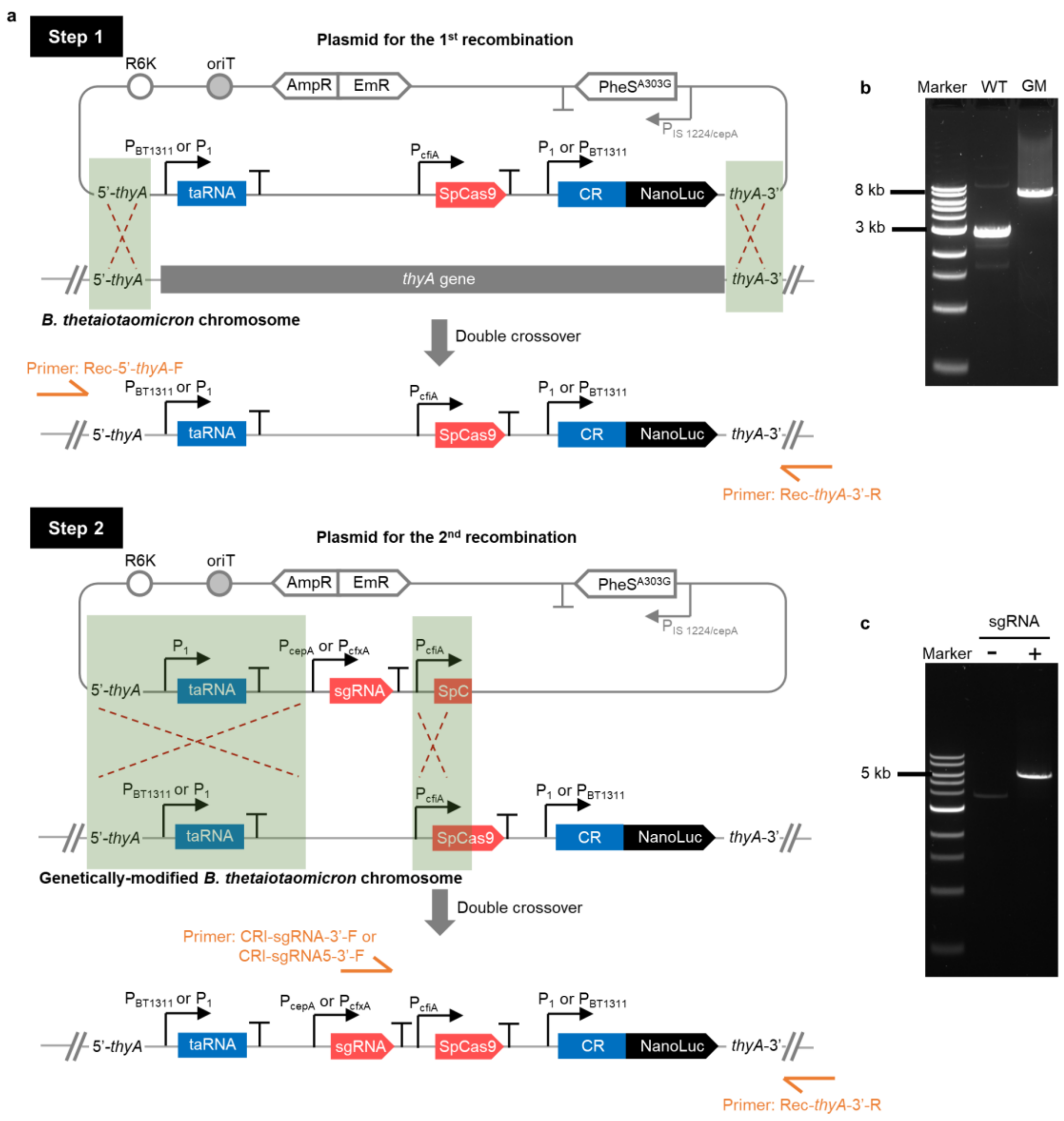


**Supplementary Fig 2 Strategy for genetic modification**. **a**, Overview of the recombination method used in this study. Step 1: Introduction of the taRNA, SpCas9, cis-repressive sequence (CR), and the NanoLuc gene onto the chromosome of wild-type (WT) *B. thetaiotaomicron*. Step 2: Introduction of sgRNA and its promoters. R6K, origin of replication; oriT, origin of transfer; AmpR, ampicillin resistance cassette; EmR, erythromycin resistance cassette; PheS$^{A303G}$, mutated α–subunit of phenylalanyl-tRNA synthetase gene. **b**, PCR identification of the integration of the gene cassette with primers

shown in (**a**). **c**, PCR identification of the integration of sgRNA. Source data are provided as a Source Data file.

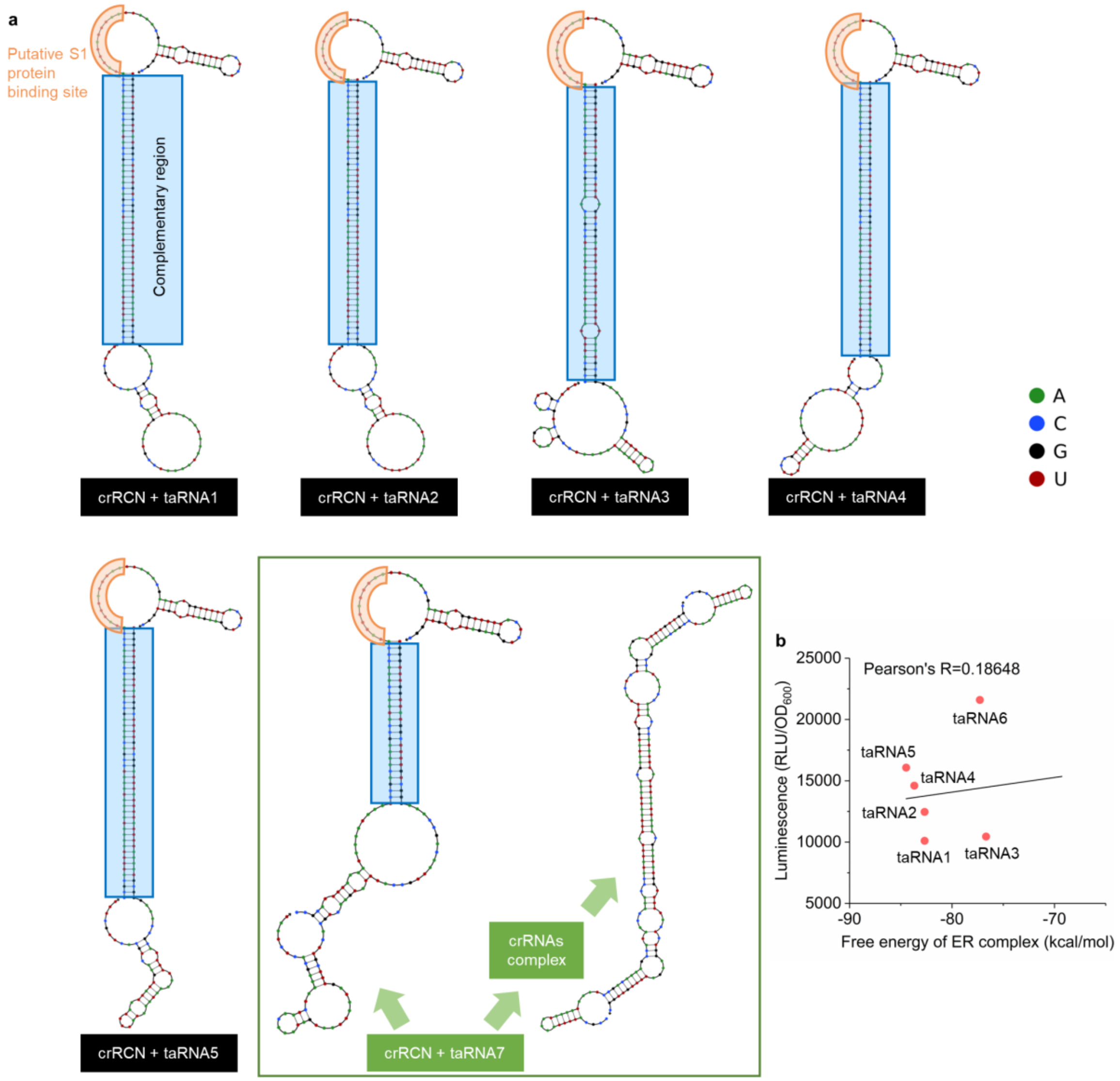


**Supplementary Fig. 3 Analysis of crRNA (crRCN)/taRNA complexes with NUPACK web server. a**, NUPACK-predicted structures of crRNA (crRCN)/taRNA complexes. **b**, Correlation between Minimum Free Energy (MRE), i.e., free energy of ER complex, and bioluminescent signal of each crRNA (crRCN)/taRNA complex: crRNA (crRCN)/taRNA1, crRNA (crRCN)/taRNA2, crRNA (crRCN)/taRNA3, crRNA (crRCN)/taRNA4, crRNA (crRCN)/taRNA5, and crRNA (crRCN)/taRNA6. Source data are provided as a Source Data file.

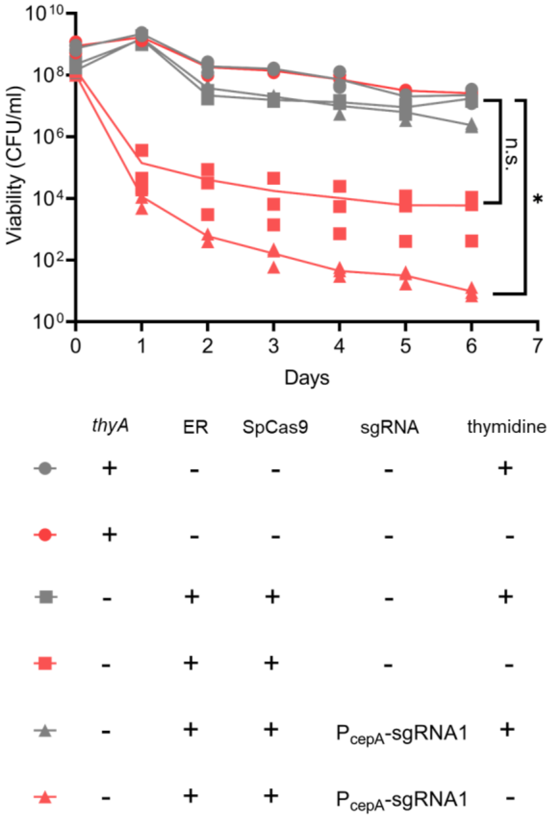


**Supplementary Fig. 4 Death rate accelerated by CRISPR Device (CD)**. Cell viability of WT strain and genetically modified strain with and without $P_{cepA}$-sgRNA1, cultured anaerobically in the presence or absence of thymidine. Each dot is a biological replicate, and lines are the values of the mean of three biological replicates (Dunn's multiple comparisons test with Kruskal-Wallis test on log-transformed data at day6, *P<0.05). n.s. represents not significant. Source data are provided as a Source Data file.

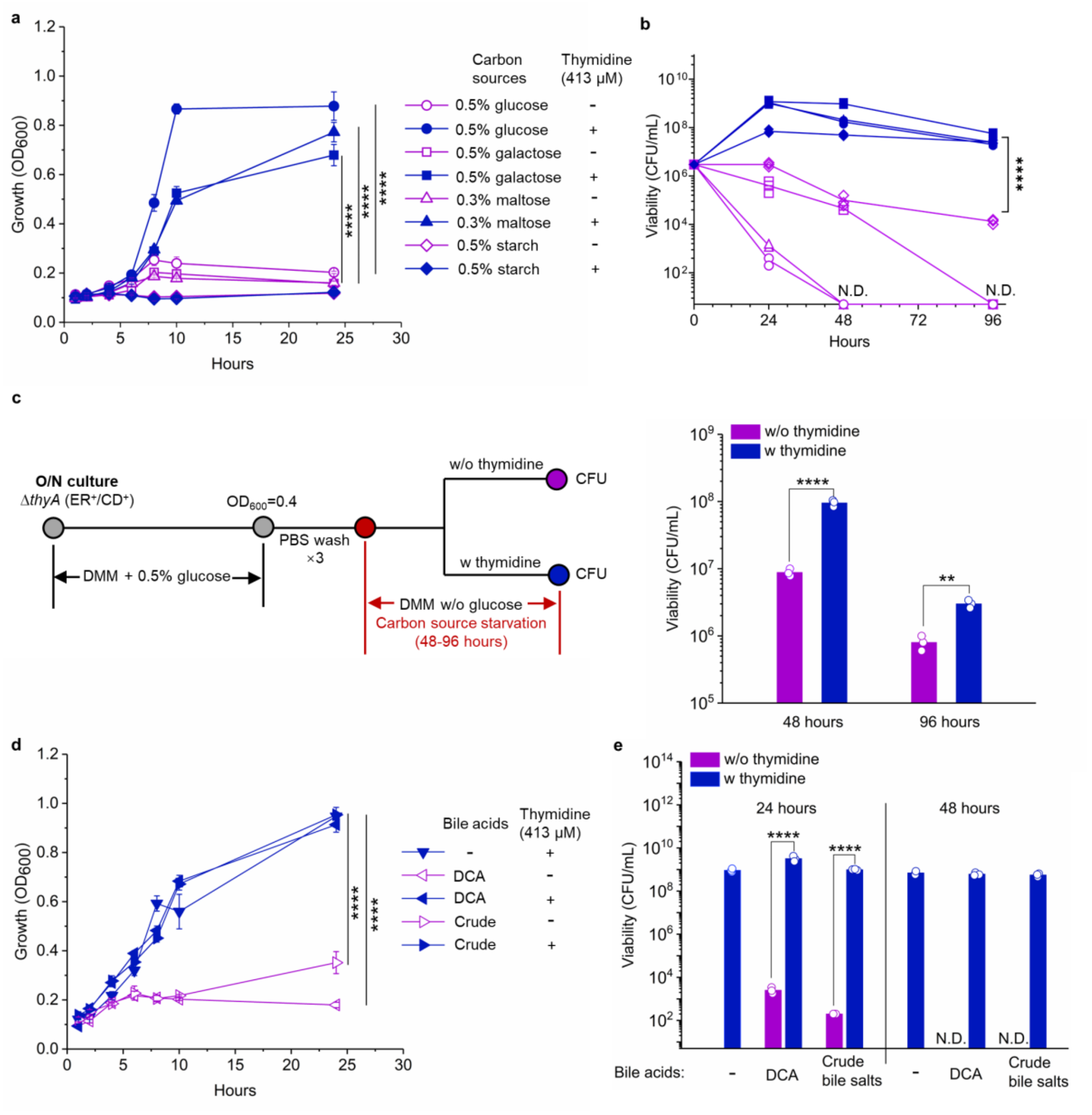


**Supplementary Fig. 5 Thymidine auxotrophy of genetically modified *B. thetaiotaomicron* in various stress conditions. a**, **b**, Growth curves (**a**) and cell viability (**b**) of *thyA*-deficient strain with $P_{cepA}$-sgRNA1 in defined minimal medium (DMM) with different carbon sources with (blue) or without (violet) thymidine. **c**, Schematic of carbon source starvation and cell viability of the engineered bacterial strain with (blue) or without (violet) thymidine. **d**, **e**, Growth curves (**d**) and cell viability (**e**) of *thyA*-deficient strain

with $P_{cepA}$-sgRNA1 in TYG medium with bile acids (62.5 uM DCA; 0.5% crude ox bile extracts) with (blue) or without (violet) thymidine.

(**a** and **d**) Each dot is the value of the mean, and error bars represent the standard deviations of three biological replicates (Tukey's multiple comparisons test with one-way analysis of variance on data at 24hrs, **** P<0.0001). (**b**) Each dot is a biological replicate, and lines are the values of the mean (Tukey's multiple comparisons test with one-way analysis of variance on log-transformed data, **** P<0.0001). The detection limit is 5 CFU/ml. (**c** and **e**) Each dot is a biological replicate, and columns are the values of the mean (two-tailed unpaired Student's t-test on log-transformed data, ** P<0.01 **** P<0.0001). The detection limit is 5 CFU/ml. N.D. represents no detection. Source data are provided as a Source Data file.

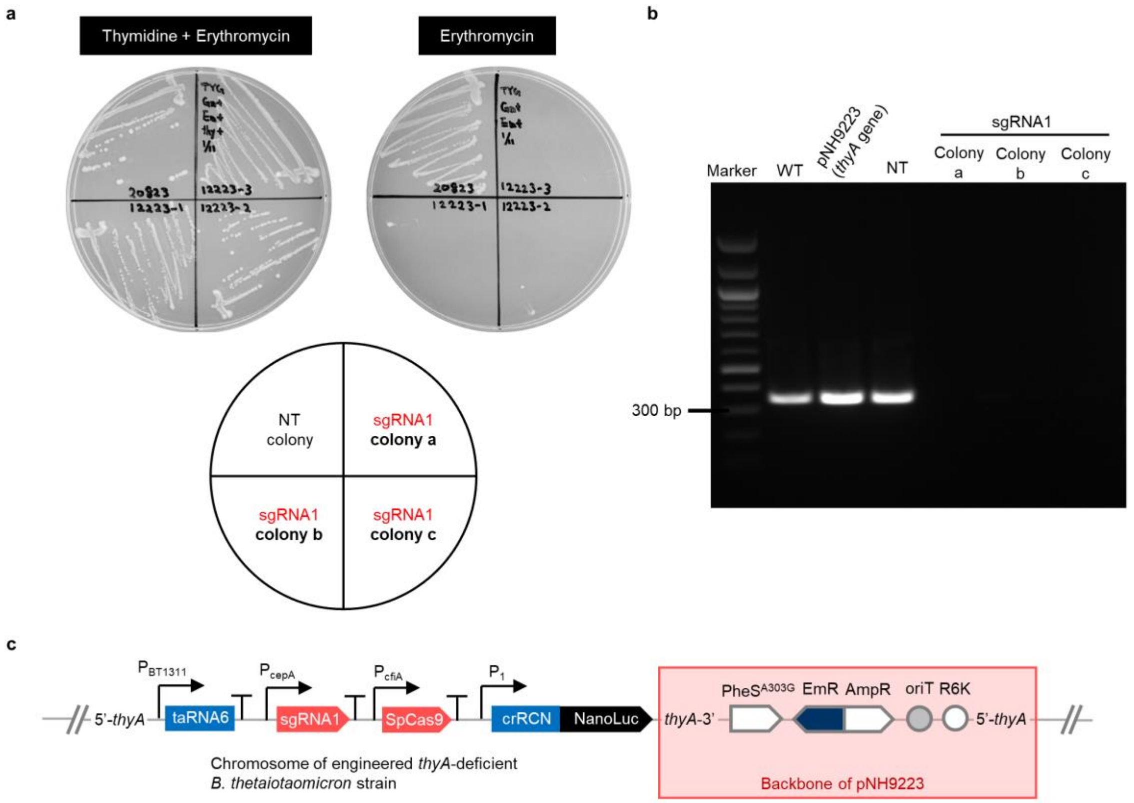


**Supplementary Fig. 6 Prevention of acquisition of *thyA* gene by genetically engineered strain**. **a**, Colonies streaked on TYG with and without thymidine in the presence of erythromycin and gentamicin. Viable *B. thetaiotaomicron* bearing sgRNA1 or NT, plated on BHIS agar plates with thymidine, erythromycin, and gentamicin after conjugation, were streaked to check thymidine auxotrophy. **b**, Amplicon patterns of transconjugants isolated from BHIS agar plates with thymidine, erythromycin, and gentamicin after conjugation. PCR was performed with primers flanking the sequence targeted by the CRISPR Device (CD) with sgRNA1 in order to detect the *thyA* gene. **c**, Schematic diagram showing integration of pNH9223 backbone into the chromosome of the engineered *thyA*-deficient *B. thetaiotaomicron* strain. Source data are provided as a Source Data file.

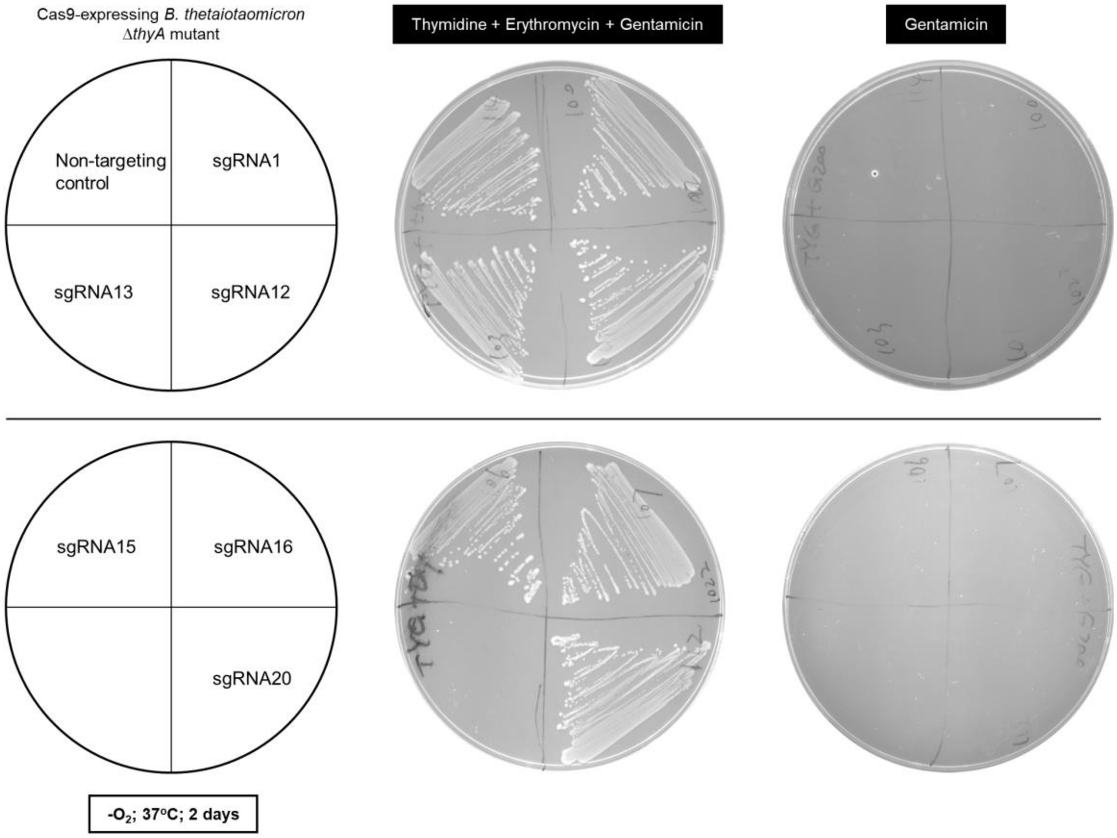


**Supplementary Fig. 7 Thymidine auxotrophy of genetically engineered strains bearing the sgRNAs and their promoter constructed by pNBU2-based technology after the first recombination**. The genetically engineered strains were streaked on gentamicin-containing TYG agar with both thymidine and erythromycin, or without either thymidine or erythromycin. Plates were incubated anaerobically at 37ºC for 2 days.

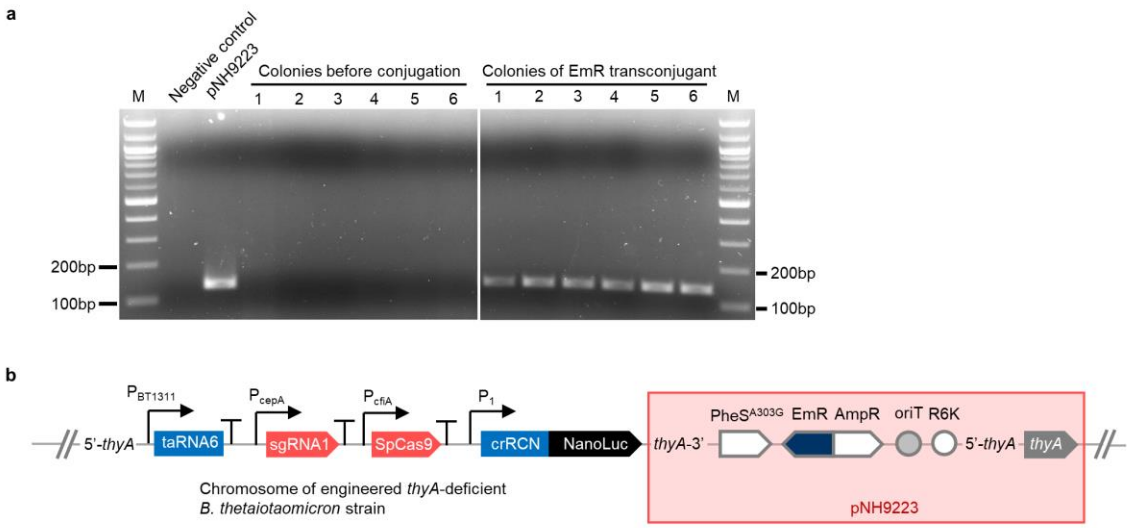


**Supplementary Fig. 8 Integration of EmR and *thyA* genes in engineered *thyA*-deficient *B. thetaiotaomicron* strain after conjugation at day 21. a**, PCR amplification of EmR gene fragment (144 bp) in engineered *B. thetaiotaomicron* strains before and after conjugation of pNH9223. **b**, Schematic diagram showing integration of pNH9223 plasmid into the chromosome of engineered *thyA*-deficient *B. thetaiotaomicron* strain. Source data are provided as a Source Data file.

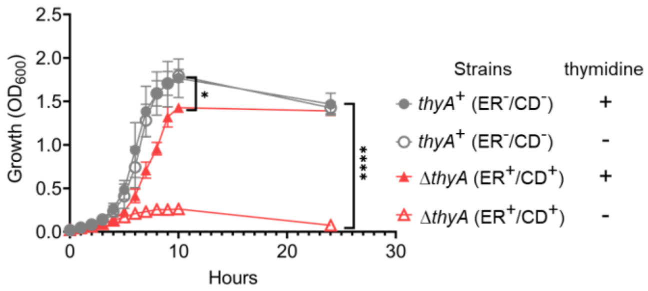


**Supplementary Fig. 9 Growth curves of *thyA*$^{+}$ (ER$^{-}$/CD$^{-}$) and Δ*thyA* (ER$^{+}$/CD$^{+}$) strains in the presence and absence of thymidine.** The cells were grown anaerobically at 37℃ for 24 hrs. 300 µl of samples were withdrawn from the cultures every hour for 10 hrs and at 24 hrs to monitor growth ($OD_{600}$). Each dot is the value of the mean, and error bars represent the standard deviations of three biological replicates made on different days (Tukey's multiple comparisons test with one-way analysis of variance on data at 10hrs and 24hrs, * P<0.05, **** P<0.0001). Source data are provided as a Source Data file.

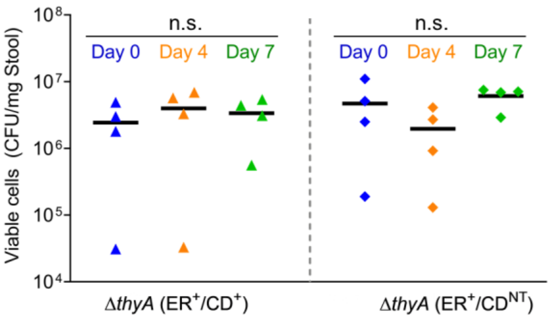


**Supplementary Fig. 10 Viability in mice feces of genetically engineered thymidine-auxotrophic *B. thetaiotaomicron* with Engineered Riboregulator (ER) and CRISPR Device (CD)**. Fecal suspensions from mice gavaged with either of Δ*thyA* ($ER^+/CD^+$) or Δ*thyA* ($ER^+/CD^{NT}$) strains but without the *thyA*$^+$ ($ER^-/CD^-$) strain were plated on selective agar plates with thymidine on the day when feces were retrieved, and after four and seven days of storage at ambient temperature in the presence of oxygen. Lines are the values of the mean of four biological replicates (Tukey's multiple comparisons test with one-way analysis of variance on log-transformed data). n.s. represents not significant. Source data are provided as a Source Data file.

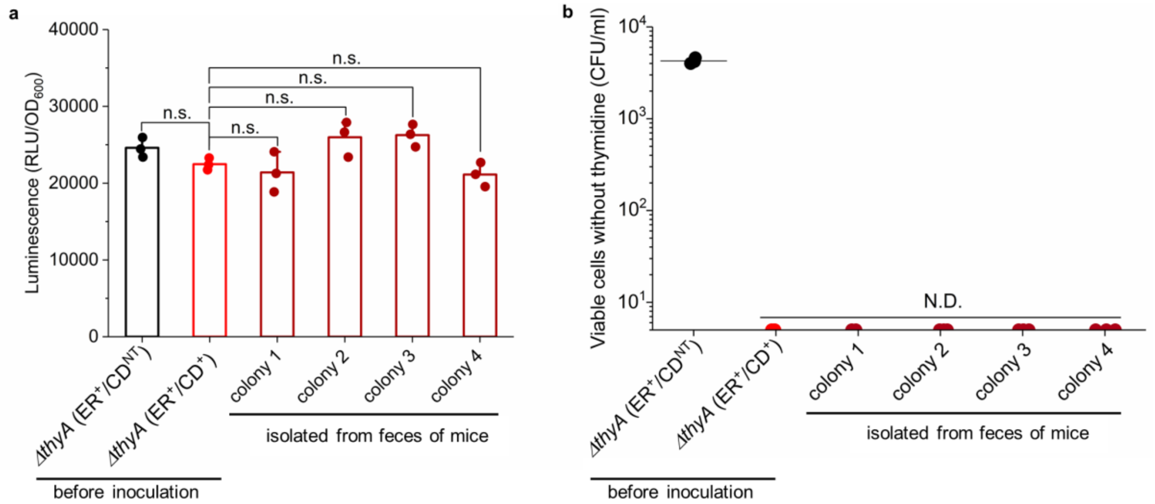


**Supplementary Fig. 11 *In vivo* genetic stability of the genetically engineered strain bearing the Engineered Riboregulator (ER) and CRISPR Device (CD)**. **a**, Outputs of NanoLuc in cells isolated from mouse feces, which were collected at day10 after administration of the genetically engineered *B. thetaiotaomicron*. Dots represent three biological replicates and columns represent the values of the mean. Error bars represent the standard deviations of the three biological replicates (Tukey's multiple comparisons test with one-way analysis of variance on data). **b**, Viable *B. thetaiotaomicron*: bacterial cells, isolated from mouse feces collected at day 10 after administration of the genetically engineered *B. thetaiotaomicron*, were conjugated with the *E. coli* donor strains which have an intact *thyA* gene on their plasmids on TYG agar plates with thymidine and then plated on TYG agar plates without thymidine. Individual dots represent three biological replicates of recipients. Black bars are the values of the mean. The detection limit is 5 CFU/ml. n.s.

represents not significant. N.D. represents no detection. Source data are provided as a Source Data file.

**Supplementary Table 1. Cis-repressive sequences (CRs) used in Figure 2.**

| Name | Sequence |
| --- | --- |
| crRCN | ccgacaaaagccataaccaaaccca |
| crR7N | tcgtttaattaaattcatattgggt |
| crR10N | tcgttaaataaaatacatattgggt |
| crR12N | tcgtataaataaatacatattgggt |

**Supplementary Table 2. Trans-activating RNAs (taRNAs) used in Figure 2.**

| Name | Description | Sequence |
|---|---|---|
| Non Sense (NS) | Non-sense trans-activating RNA | taatacccaaatccaggaggtgattggtagtggtggttaatgaaaattaacttactactaccatatatctctaga |
| taRNA1 | trans-activating RNA | tgggtttggttatggcttttgtcggatttattttattttaaaatgcgggaaaccgcatttttaaaataaataaaatccgtaa |
| taRNA2 | trans-activating RNA | tgggtttggttatggcttttgtcggatttattttattttaaaatgcgggaaaccgcatttttaaaataaatattttccgtaa |
| taRNA3 | trans-activating RNA | tgggtttggttatggctttagtcggatttattttattttaaatgcgggaaaccgcatttataaaataaataaaatccgtaa |
| taRNA4 | trans-activating RNA | tgggtttggttatggcttttgtcggatttattttattttaaaatgcgggaaaccgcatttttaaaataaatattttccgtat |
| taRNA5 | trans-activating RNA | tgggtttggttatggcttttgtcggatttattttattttaaaatgcgggaaaccgcatttttaaaataaatattttaattat |
| taRNA6 | trans-activating RNA | tgggtttggttatggctttagtcggatttattttattttaaatgcgggaaaccgcatttataaaataaatattttaattat |
| taRNA7 | trans-activating RNA | tgggtttggttatggcttttgtcgggaaaccgacaattgccattta |

Sequences complementary to crRNA are highlighted in blue.

**Supplementary Table 3. Single guide RNAs (sgRNAs) used in experiments.**

| Name | Description | Figure | Sequence | PAM |
|---|---|---|---|---|
| Non-targeting control (NT) | Non-targeting single guide RNA | 4, 5, 7, S6, S7, S10, S11 | attctctagactttacttga | None |
| sgRNA1 | Single guide RNA | 3, 4, 5, 6, 7, S1, S4, S5, S6, S7, S8, S9, S10, S11 | acgcatctggaacgaatggg | CGG |
| sgRNA12 | Single guide RNA | 4, S7 | aggcgctgacaatgatacgg | CGG |
| sgRNA13 | Single guide RNA | 4, S7 | atgcgtttcaacctcgacga | GGG |
| sgRNA15 | Single guide RNA | 4, S7 | actgagaaaagtgaccgtac | AGG |
| sgRNA16 | Single guide RNA | 4, S7 | agttttacgtggcagacggt | CGG |
| sgRNA20 | Single guide RNA | 4, S7 | gcctcaaatgaaaattaatc | CGG |

**Supplementary Table 4. Genetic parts used in experiments.**

| Name | Description (Source) | Sequence |
|---|---|---|
| AmpR | Ampicillin-resistance cassette (Cell Syst. 2015; 1(1): 62-71.) | atgagtattcaacatttccgtgtcgcccttattcccttttttgcggcattttgccttcctgtttttgctcacccagaaacgctggtgaaagtaaaagatgctgaagatcagttgggtgcacgagtgggttacatcgaactggatctcaacagcggtaagatccttgagagttttcgccccgaagaacgttttccaatgatgagcacttttaaagttctgctatgtggcgcggtattatcccgtattgacgccgggcaagagcaactcggtcgccgcatacactattctcagaatgacttggttgagtactcaccagtcacagaaaagcatcttacggatggcatgacagtaagagaattatgcagtgctgccataaccatgagtgataacactgcggccaacttacttctgacaacgatcggaggaccgaaggagctaaccgcttttttgcacaacatgggggatcatgtaactcgccttgatcgttgggaaccggagctgaatgaagccataccaaacgacgagcgtgacaccacgatgcctgtagcaatggcaacaacgttgcgcaaactattaactggcgaactacttactctagcttcccggcaacaattaatagactggatggaggcggataaagttgcaggaccacttctgcgctcggcccttccggctggctggtttattgctgataaatctggagccggtgagcgtgggtctcgcggtatcattgcagcactggggccagatggtaagccctcccgtatcgtagttatctacacgacggggagtcaggcaactatggatgaacgaaatagacagatcgctgagataggtgcctcactgattaagcattggtaa |
| EmR | Erythromycin-resistance cassette (Cell Syst. 2015; 1(1): 62-71) | atgaacaaagtaaatataaaagatagtcaaaattttattacttcaaaatatcacatagaaaaaataatgaattgcataagtttagatgaaaaagataacatctttgaaataggtgcagggaaaggtcattttactgctggattggtaaagagatgtaattttgtaacggcgatagaaattgattctaaattatgtgaggtaactcgtaataagctcttaaattatcctaactatcaaatagtaaatgatgatatactgaaatttacatttcctagccacaatccatataaaatatttggcagcataccttacaacataagcacaaatataattcgaaaaattgtttttgaaagttcagccacaataagttatttaatagtggaatatggttttgctaaaatgttattagatacaaacagatcactagcattgctgttaatggcagaggtagatatttctatattagcaaaaattcctaggtattatttccatccaaaacctaaagtggatagcacattaattgtattaaaaagaaagccagcaaaaatggcatttaaagagagaaaaaaatatgaaacttttgtaatgaaatgggttaacaaagagtacgaaaaactgtttacaaaaaatcaatttaataaagctttaaaacatgcgagaatatatgatataaacaatattagtttcgaacaatttgtatcgctatttaatagttataaaatatttaacggctaa |
| R6K | Origin of replication (Cell Syst. 2015; 1(1): 62-71) | gatctgaagatcagcagttcaacctgttgatagtacgtactaagctctcatgtttcacgtactaagctctcatgtttaacgtactaagctctcatgtttaacgaactaaaccctcatggctaacgtactaagctctcatggctaacgtactaagctctcatgtttcacgtactaagctctcatgtttgaacaataaaattaatataaatcagcaacttaaatagcctctaaggttttaagttttataagaaaaaaaagaatatataaggctttaaagcttttaaggtttaacggttgtggacaacaagccagggatgtaacgcactgagaagcccttagagcctctcaaagcaattttgagtgacacaggaacacttaacggctgaca |
| RP4 | Origin of transfer (Cell Syst. 2015; 1(1): 62-71) | ccggccagcctcgcagagcaggattcccgttgagcaccgccaggtgcgaataagggacagtgaagaaggaacacccgctcgcgggtgggcctacttcacctatcctgccc |

| | | |
|---|---|---|
| $PheS^{A303G}$ | Mutated alpha subunit of Bacteroides phenylalanyl tRNA synthetase (Anaerobe. 2016; 42: 81-88.) | atgatagctaagattaatcaacttcttgaagaggtgggggccttgaaagccg<br>ccaatgccgaagaactcgaagttctgcgcatcaaatacctcagcaagaaa<br>ggagccatcaatgacttgatggcagatttccgcaatgtggctgccgaacag<br>aaaaaagaagtcggcatgaagctcaacgagctgaaaacaaaagcaca<br>agaaaaaatcaacgcactgaaagaacagttcgacaaccaggacaacgg<br>acaggacgatctcgacctgacccgttcggcttatcccgtggagctgggcac<br>acgccatccgctttccattgtgcgcaacgaaatcattgacatctttgcccgtct<br>gggattcaacattgccgaaggtccggaaatagaagatgactggcatgtgtt<br>ctccgcactcaactttgccgaagaccatccggcacgagacatgcaagaca<br>cattcttcatcgaatcccatcccgacgtactactgcgcacacacacctcatcg<br>gtacagagccgtgtgatggaggtttcgcaaccacctatccgtatcatctgtcc<br>gggacgcgtttaccgtaacgaagctatcagctatcgtgcacactgtttcttcca<br>ccaggtagaagcgctgtacgtagaccgcaatgtatctttcaccgacctgaa<br>acaagtgttgctactcttcgccaaagagatgttcggtgccgatacgaagattc<br>gcctgcgtccgtcgtacttcccgttcacagaacccagcgccgaaatggatat<br>cagctgtaacatctgcggcggaaaaggctgccccttctgcaaacacaccg<br>gctgggtcgaaatcttgggttgcggaatggtagacccgaacgtacttgatgc<br>caacggaatagacagcaaagtgtatagcggatatggactgggtatgggtat<br>cgaacgcatcacaaacctgaaatatcaggtgaaagacctccgcatgttctc<br>cgaaaacgatacacgtttcctgaaggagttcgaagcggcctattaa |
| $P_{IS\ 1224/cepA}$ + RBS | Constitutive promoter + RBS (Anaerobe. 2016; 42: 81-88.) | cctttgaaaagagaagaagccgtgttgctgttcaaactggtcaatgacttcca<br>ggaaaggacatcactcatcatcacggcaaacaaggcactcacccgttggc<br>tggaaacattggaggatgaagcggtcacagccgccttgcttgacaggctgc<br>tctactgctgcgagattatcaggctcggaggaacaagctatcgcatgcaaa<br>acaggaaaacaatttttagcaaccaaaacacggatataggcacgtaaaa<br>agagttaaggaaagtgaagcatcttcgatgctggagtgggatatactaaatt<br>acctaaaaaagtggcgcacaaatttgcgcgccacaattattattcatacctttt<br>gtggaccgtattacaaagaacccaatcatat |
| $P_{BT1311}$ | Constitutive promoter (Cell Syst. 2015; 1(1): 62-71) | tgatctggaagaagcaatgaaagctgctgttaagtctccgaatcaggtattgt<br>tcctgacaggtgtattcccatccggtaaacgcggatactttgcagttgatctga<br>ctcaggaataaattataaattaaggtaagaagattgtaggataagctaatga<br>aatagaaaaaggatgccgtcacacaacttgtcggcattctttttgttttattagt<br>tgaaaatatagtgaaaaagttgcctaaatatgtatgttaacaaattatttgtcgt<br>aactttgcactc |
| $P_1$ | Constitutive promoter (Cell Syst. 2015; 1(1): 62-71) | gataaagtttggaagataaagctaaaagttcttatctttgcagt |
| $P_{cfiA}$ + RBS | Constitutive promoter + RBS (Cell Syst. 2015; 1(1): 62-71) | ggagtgagcttctcggattttatttgtatttttgccatgcctgatgaggttttgtttga<br>ttattttttgcaacactaagttaagtgaatcctctgacatggcaaaatcctgag<br>caactttttgttgctcaggtacttaaaaaaaatattttataatagtgttgcggaatt<br>aaggtaaaagaataaa |
| $P_{cfxA}$ | Constitutive promoter (Cell Syst. 2015; 1(1): 62-71) | tacaaagaaaattcgacaaactgttatttttctatctatttatttgggtgggaaac<br>tttagttatgtacctttgtcggc |

| | | |
|---|---|---|
| $P_{cepA}$ | Constitutive promoter<br>(Cell Syst. 2015; 1(1): 62-71) | caaatttgcgcgccacaattattattcatacctttgtgg |
| rpiL* | RBS<br>(Cell Syst. 2015; 1(1): 62-71) | ccgcattttaaaataaaataaattatttatttaattaaacgaat |
| 5'-*thyA* | Upstream of *thyA* gene of *B. thetaiotaomicron*<br>(This work) | tctatagccaatgcgctgttcgacctgctggccgaaaaagtgaaagaaggc<br>gtggaagtacgagccatgttcgatgcgttcggcaactggtcgaacaataaa<br>ccgctcaagaagagacatctcaaaaaaatacgagagcagggaatcgag<br>attgtcaagttcgacccgttcacctttccctacatcaatcatgcggcacaccgt<br>gatcaccgcaaaatagccgtcatcgacggtgaagtcgcctatacgggagg<br>aatgaacatcgccgactactacatcaacggactccccaaaatcggtacatg<br>gcgtgatatgcacatgagaatagaaggcgatgccgtcaatgacctgcaag<br>aaatatttcttaccatatggaacaaggagactaaacaaaatattggcggcg<br>aagcatacttccccaagcacaaggagcagtcggacagcaccaatgtagtc<br>gtcgccatcgtagaccgcacaccgaagaaaaacagccgtatgctcagtca<br>tgcttatgccatgtcgatctattcggcacagaaaaacgtgcacatcgtcaacc<br>cgtattttgtgcccacatcctccatcaacaaggcgcttcaacgcaccatcga<br>acgaggtgtggatgtcacgattatggtttcttctgcctccgacatcccgtttactc<br>cggacgccgccctctacaaacttcacaaactgatgaaaagggggccac<br>cgtctatatgtacaatggcggtttccaccattccaaaatcatgatggtcgacg<br>atattttctgcacggtaggcaccgcaaacctgaacagccgcagcctccggt<br>acgactacgagacgaacgctttcatctttaacaaggaaattacaggcgaac<br>tgaacgaaatgtttcggaacgatatagaacactgtacgcagctaacaccgg<br>aattctggaaaaagcgctcaccgtggaagaagttcgtcggatggtttgccaa<br>tttattcacgccatttttgtaattttgcatcgcaccaacttattggaaaa |
| *thyA*-3' | Downstream of *thyA* gene of *B. thetaiotaomicron*<br>(This work) | ccacatattgccggaatagtagcggtataaaaccatgagtaaaatatcaatt<br>attgccgccgtagaccgccgaatggcgatcggctttcagaataaactgctttt<br>ctggttgcccaacgacttgaaacgcttcaaggcactgactaccggaaacac<br>catcataatgggaagaaaaaccttcgaatcgctgccgaaaggtgcgttgcc<br>caatcgcagaaacgttgtgttatccacccgtccggatacggtatgtcccggtg<br>cggaagtcttcccgtcgctggaagtcgccctgcaaagctgcaaggaagat<br>gagcacgtctatataataggtggcgcaagcgtctaccagcaggcacttcctc<br>ttgccgacgaactttgtctgacggaaataaatgacgttgctcccgaagccga<br>tgccttctttccggaagtatctccggctcaatggcacgaaaaaagcagaga<br>agctcatcctgtggatgagaaacatctctgcccgtatgcttttgtagattacgtg<br>aaacagtaagctattttattctgtacgattaatcttcgtcttcaatgtcgaccata<br>atcggcatctgccgctttatggcttcatggaaagagatcaatgtctccgtacg<br>cgccaaccccaaaggttgcaatttatcatgtatgatactcagcaaatggtgat<br>tgttcttagcgtaaatcttgataaacatatcatatttaccagtagtgaaatgaca<br>ctctactacttccggaatggcttccaaagctctcgttacagaatcaaaggattc<br>cggatctttcagatatataccaatataagcacaagtttcatatcctatcttctca<br>ggatcgatcacatattccgatcctttcaatattcccagattagtcaacttctgaa<br>tgcgctggtggatagccgccccggaaacgttacacgctcttgctacttccaa<br>a |
| CR | Cis-Represive sequence<br>(This work) | See Supplementary Table 1 |

| | | |
|---|---|---|
| taRNA | trans-activating RNA<br>(This work) | See Supplementary Table 2 |
| SpCas9 | Cas9 gene of *Streptococcus pyogenes* modified to work in *B. thetaiotaomicron*<br>(This work) | atggataagaaatactcaataggcttagatatcggcacaaatagcgtcgga<br>tgggcggtgatcactgatgaatataaggttccgtctaaaaagttcaaggttct<br>gggaaatacagaccgccacagtatcaaaaaaaatcttataggggctctttta<br>tttgacagtggagagacagcggaagcgactcgtctcaaacggacagctcg<br>tagaaggtatacacgtcggaagaatcgtatttgttatctacaggagatttttca<br>aatgagatggcgaaagtagatgatagtttctttcatcgacttgaagagtcttttt<br>ggtggaagaagacaagaagcatgaacgtcatcctatttttggaaatatagta<br>gatgaagttgcttatcatgagaaatatccaactatctatcatctgcgaaaaaa<br>attggtagattctactgataaagcggatttgcgcttaatctatttggccttagcgc<br>atatgattaagtttcgtggtcatttttgattgagggagatttaaatcctgataata<br>gtgatgtggacaaactatttatccagttggtacaaacctacaatcaattatttg<br>aagaaaaccctattaacgcaagtggagtagatgctaaagcgattctttctgc<br>acgattgagtaaatcaagacgattagaaaatctcattgctcagctccccggt<br>gagaagaaaaatggcttatttgggaatctcattgctttgtcattgggtttgaccc<br>ctaattttaaatcaaattttgatttggcagaagatgctaaattacagctttcaaa<br>agatacttacgatgatgatttagataatttattggcgcaaattggagatcaatat<br>gctgatttgtttttggcagctaagaatttatcagatgctattttactttcagatatcc<br>taagagtaaatactgaaataactaaggctcccctatcagcttcaatgattaaa<br>cgctacgatgaacatcatcaagacttgactcttttaaaagctttagttcgacaa<br>caacttccagaaaagtataaagaaatcttttttgatcaatcaaaaaacggat<br>atgcaggttatattgatgggggagctagccaagaagaattttataaatttatca<br>aaccaattttagaaaaaatggatggtactgaggaattattggtgaaactaaa<br>tcgtgaagatttgctgcgcaagcaacggacctttgacaacggctctattcccc<br>atcaaattcacttgggtgagctgcatgctattttgagaagacaagaagactttt<br>atccatttttaaaagacaatcgtgagaagattgaaaaaatcttgacttttcgaa<br>ttccttattatgttggtccattggcgcgtggcaatagtcgttttgcatggatgactc<br>ggaagtctgaagaaacaattaccccatggaattttgaagaagttgtcgataa<br>aggtgcttcagctcaatcatttattgaacgcatgacaaactttgataaaaatctt<br>ccaaatgaaaaagtactaccaaaacatagtttgctttatgagtattttacggttt<br>ataacgaattgacaaaggtcaaatatgttactgaaggaatgcgaaaacca<br>gcatttctttcaggtgaacagaagaaagccattgttgatttactcttcaaaaca<br>aatcgaaaagtaaccgttaagcaattaaaagaagattatttcaaaaaaata<br>gaatgttttgatagtgttgaaatttcaggagttgaagatagatttaatgcttcatt<br>aggtacctaccatgatttgctaaaaattattaaagataaagatttttttggataat<br>gaagaaaatgaagatatcttagaggatattgttttaacattgaccttatttgaa<br>gatagggagatgattgaggaaagacttaaaacatatgctcacctctttgatg<br>ataaggtgatgaaacagcttaaacgtcgccgttatactggttggggacgtttg<br>tctcgaaaattgattaatggtattagggataagcaatctggcaaaacaatatt<br>agatttttttgaaatcagatggttttgccaatcgcaattttatgcagctgatccatg<br>atgatagtttgacatttaaagaagacattcaaaaagcacaagtgtctggaca<br>aggcgatagtttacatgaacatattgcaaatttagctggtagccctgctattaa<br>aaaaggtattttacagactgtaaaagttgttgatgaattggtcaaagtaatgg<br>ggcggcataagccagaaaatatcgttattgaaatggcacgtgaaaatcag<br>acaactcaaaagggccagaaaaattcgcgagagcgtatgaaacgaatcg<br>aagaaggtatcaaagaattaggaagtcagattcttaaagagcatcctgttga<br>aaatactcaattgcaaaatgaaaagctctatctctattatctccaaaatggaa<br>gagacatgtatgtggaccaagaattagatattaatcgtttaagtgattatgatg<br>tcgatcacattgttccacaaagtttccttaaagacgattcaatagacaataag |

| | | |
|---|---|---|
| | | gtcttaacgcgttctgataaaaatcgtggtaaatcggataacgttccaagtga<br>agaagtagtcaaaaagatgaaaaactattggagacaacttctaaacgcca<br>agttaatcactcaacgtaagtttgataatttaacgaaagctgaacgtggaggt<br>ttgagtgaacttgataaagctggttttatcaaacgccaattggttgaaactcgc<br>caaatcactaagcatgtggcacaaattttggatagtcgcatgaatactaaat<br>acgatgaaaatgataaacttattcgagaggttaaagtgattaccttaaaatct<br>aaattagtttctgacttccgaaaagatttccaattctataaagtacgtgagatta<br>acaattaccatcatgcccatgatgcgtatctaaatgccgtcgttggaactgctt<br>tgattaagaaatatccaaaacttgaatcggagtttgtctatggtgattataaagt<br>ttatgatgttcgtaaaatgattgctaagtctgagcaagaaataggcaaagca<br>accgcaaaatatttcttttactctaatatcatgaacttcttcaaaacagaaatta<br>cacttgcaaatggagagattcgcaaacgccctctaatcgaaactaatgggg<br>aaactggagaaattgtctgggataaagggcgagattttgccacagtgcgca<br>aagtattgtccatgccccaagtcaatattgtcaagaaaacagaagtacaga<br>caggcggattctccaaggagtcaattttaccaaaaagaaattcggacaagc<br>ttattgctcgtaaaaaagactgggatccaaaaaaatatggtggttttgatagtc<br>caacggtagcttattcagtcctagtggttgctaaggtggaaaaagggaaatc<br>gaagaagttaaaatccgttaaagagttactagggatcacaattatggaaag<br>aagttcctttgaaaaaatccgattgacttttagaagctaaaggatataagg<br>aagttaaaaaagacttaatcattaaactacctaaatatagtctttttgagttaga<br>aaacggtcgtaaacggatgctggctagtgccggagaattacaaaaagga<br>aatgagctggctctgccaagcaaatatgtgaatttttatatttagctagtcatta<br>tgaaaagttgaagggtagtccagaagataacgaacaaaaacaattgtttgt<br>ggagcagcataagcattatttagatgagattattgagcaaatcagtgaattttc<br>taagcgtgttattttagcagatgccaatttagataaagttcttagtgcatataac<br>aaacatagagacaaaccaatacgtgaacaagcagaaaatattattcattta<br>tttacgttgacgaatcttggagctcccgctgcttttaaatattttgatacaacaatt<br>gatcgtaaacgatatacgtctacaaaagaagttttagatgccactcttatccat<br>caatccatcactggtctttatgaaacacgcattgatttgagtcagctaggaggt<br>gac |
| NanoLuc | NanoLuc gene<br>(Cell Syst. 2015;<br>1(1): 62-71) | atggtttttactctggaagattttgttggcgattggcgtcagaccgcgggttata<br>atttggatcaagtcctggaacagggtggcgtaagctctctgttccagaacctg<br>ggtgtgagcgtgacgccgattcagcgcatcgttctgtccggcgagaacggt<br>ctgaaaattgatattcatgtgatcatcccgtacgaaggcctgagcggtgacc<br>aaatgggtcaaatcgagaaaatctttaaagtcgtctacccagttgacgatca<br>ccacttcaaggttatcttgcattacggtacgctggtgattgatggtgtgacccc<br>gaatatgattgactatttcggccgtccgtatgaaggcattgccgtttttgacggt<br>aaaaagatcaccgtcaccggtaccctgtggaatggcaataagattattgac<br>gagcgtctgattaacccggacggcagcctgctgttccgcgtgaccatcaac<br>ggtgtcacgggttggcgtctgtgcgagcgcatcctggcataa |
| sgRNA1-<br>upstream | Upstream of<br>sgRNA<br>(This work) | tctatagccaatgcgctgttcgacctgctggccgaaaaagtgaaagaaggc<br>gtggaagtacgagccatgttcgatgcgttcggcaactggtcgaacaataaa<br>ccgctcaagaagagacatctcaaaaaaatacgagagcagggaatcgag<br>attgtcaagttcgacccgttcacctttccctacatcaatcatgcggcacaccgt<br>gatcaccgcaaaatagccgtcatcgacggtgaagtcgcctatacgggagg<br>aatgaacatcgccgactactacatcaacggactccccaaaatcggtacatg<br>gcgtgatatgcacatgagaatagaaggcgatgccgtcaatgacctgcaag<br>aaatatttcttaccatatggaacaaggagactaaacaaaatattggcggcg<br>aagcatacttccccaagcacaaggagcagtcggacagcaccaatgtagtc<br>gtcgccatcgtagaccgcacaccgaagaaaaacagccgtatgctcagtca |

| | | |
|---|---|---|
| | | tgcttatgccatgtcgatctattcggcacagaaaaacgtgcacatcgtcaacc cgtattttgtgcccacatcctccatcaacaaggcgcttcaacgcaccatcga acgaggtgtggatgtcacgattatggtttcttctgcctccgacatcccgtttactc cggacgccgccctctacaaacttcacaaactgatgaaaagggggccac cgtctatatgtacaatggcggtttccaccattccaaaatcatgatggtcgacg atattttctgcacggtaggcaccgcaaacctgaacagccgcagcctccggt acgactacgagacgaacgctttcatctttaacaaggaaattacaggcgaac tgaacgaaatgtttcggaacgatatagaacactgtacgcagctaacaccgg aattctggaaaaagcgctcaccgtggaagaagttcgtcggatggtttgccaa tttattcacgccatttttgtaattttgcatcgcaccaacttattggaaaaagcacg tcgtctcagactgaggtcactgagagtagctgatctggaagaagcaatgaa agctgctgttaagtctccgaatcaggtattgttcctgacaggtgtattcccatcc ggtaaacgcggatactttgcagttgatctgactcaggaataaattataaatta aggtaagaagattgtaggataagctaatgaaatagaaaaaggatgccgtc acacaacttgtcggcattctttttgttttattagttgaaaatatagtgaaaaagtt gcctaaatatgtatgttaacaaattatttgtcgtaactttgcactctgggtttggtt atggctttagtcggatttattttattttaaatgcgggaaaccgcatttataaaata aatattttaattattgaactgcacttgctttgataattaatgataaacaatctaaa agcactctaatcgttatcggagtgcttttagattactaatcaaattgcttctacta attgcctatcttccagtgatggaacagcatttgtgcattggctgcaacaacgct aagatgctgg |
| sgRNA1-downstream | Downstream of sgRNA (This work) | ccacgtgctaaggagtgagcttctcggattttatttgtatttttgccatgcctgatg aggttttgtttgattattttttgcaacactaagttaagtgaatcctctgacatggc aaaatcctgagcaactttttgttgctcaggtacttaaaaaaaatattttataata gtgttgcggaattaaggtaaaagaataaaatggataagaaatactcaatag gcttagatatcggcacaaatagcgtcggatgggcggtgatcactgatgaat ataaggttccgtctaaaaagttcaaggttctgggaaatacagaccgccaca gtatcaaaaaaaatcttataggggctcttttatttgacagtggagagacagcg gaagcgactcgtctcaaacggacagctcgtagaaggtatacacgtcggaa gaatcgtatttgttatctacaggagatttttcaaatgagatggcgaaagtagat gatagtttctttcatcgacttgaagagtctttttggtggaagaagacaagaag catgaacgtcatcctatttttggaaatatagtagatgaagttgcttatcatgaga aatatccaactatctatcatctgcgaaaaaaattggtagattctactgataaa gcggatttgcgcttaatctatttggccttagcgcatatgattaagtttcgtggtcat tttttgattgagggagatttaaatcctgataatagtgatgtggacaaactatttat ccagttggtacaaacctacaatcaattatttgaagaaaaccctattaacgca agtggagtagatgctaaagcgattctttctgcacgattgagtaaatcaagac gattagaaaatctcattgctcagctccccggtgagaagaaaaatggcttattt gggaatctcattgctttgtcattgggtttgacccctaattttaaatcaaattttgatt tggcagaagatgctaaattacagctttcaaaagatacttacgatgatgattta gataatttattggcgcaaattggagatcaatatgctgatttgtttttggcagctaa gaatttatcagatgctattttactttcagatatcctaagagtaaatactgaaata actaaggctcccctatcagcttcaatgattaaacgctacgatgaacatcatca agacttgactcttttaaaagctttagttcgacaacaacttccagaaaagtataa agaaatctttttgatcaatcaaaaaacggatatgcaggttatattgatgggg agctagccaagaagaattttataaatttatcaaaccaatttagaaaaaatgg atggtactgaggaattattggtgaaactaaatcgtgaagatttgctgcgcaag c |
| sgRNA | Single guide RNA (This work) | See Supplementary Table 3 |

| | | |
|---|---|---|
| gRNA scaffold | Guide RNA scaffold<br>(Cell Syst. 2015; 1(1): 62-71) | gttttagagctagaaatagcaagttaaaataaggctagtccgttatcaacttg<br>aaaaagtggcaccgagtcggtgc |
| *thyA* | *thyA* gene<br>(Science. 2003; 299(5615): 2074-6.) | atgaaacaatatttagatttactcaatcgcgtattaactgaaggaactgagaa<br>aagtgaccgtacaggaaccggaacgatcagtgtgttcggacatcagatgc<br>gtttcaacctcgacgagggtttcccgtgtctgaccaccaaaaaactgcatctg<br>aaatcaatcatctacgagttactctggtttctgcaaggtgatacgaacgcgaa<br>atatctgcaagaacacggtgtacgcatctggaacgaatgggcggacgaga<br>acggtgatttagggcatatctatggctatcagtggcgttcgtggcccgactac<br>gacggcggattcatcgaccagatcagcgaagcggtagagacgatcaagc<br>acaatcccgactcccgccgtatcattgtcagcgcctggaatgtagccgattta<br>aagaatatgaacctgcctccctgtcatgccttcttccagttttacgtggcagac<br>ggtcggctgagcctgcaactttaccagcgcagcgcggatatatttctcggag<br>tcccgttcaatatcgcatcatatgcactgctgctacaaatgatggcgcaggtg<br>acaggactgaaagcgggtgaatttatccatacacttggcgatgcccatatct<br>atctgaaccacttggatcaggtcaaattgcagcttagccgcgaaccacgcg<br>cattgcctcaaatgaaaattaatccggatgtgaagagtatctatgacttccagt<br>tcgaagacttcgaactggtgaactacgacccgcatccacatattgccggaat<br>agtagcggtataa |
| Mutated *thyA* | Mutated *thyA* gene<br>(This work) | atgaaacaatatttagatttactcaatcgcgtattaactgaaggaactgagaa<br>aagtgaccgtacaggaaccggaacgatcagtgtgttcggacatcagatgc<br>gtttcaacctcgacgagggtttcccgtgtctgaccaccaaaaaactgcatctg<br>aaatcaatcatctacgagttactctggtttctgcaaggtgatacgaacgcgaa<br>atatctgcaagaacacggtgtgcgtatttggaatgagtgggcggacgagaa<br>cggtgatttagggcatatctatggctatcagtggcgttcgtggcccgactacg<br>acggcggattcatcgaccagatcagcgaagcggtagagacgatcaagca<br>caatcccgactcccgccgtatcattgtcagcgcctggaatgtagccgatttaa<br>agaatatgaacctgcctccctgtcatgccttcttccagttttacgtggcagacg<br>gtcggctgagcctgcaactttaccagcgcagcgcggatatatttctcggagtc<br>ccgttcaatatcgcatcatatgcactgctgctacaaatgatggcgcaggtga<br>caggactgaaagcgggtgaatttatccatacacttggcgatgcccatatctat<br>ctgaaccacttggatcaggtcaaattgcagcttagccgcgaaccacgcgca<br>ttgcctcaaatgaaaattaatccggatgtgaagagtatctatgacttccagttc<br>gaagacttcgaactggtgaactacgacccgcatccacatattgccggaata<br>gtagcggtataa |
| NBU2 integrase | *intN2* tyrosine integrase gene to integrate plasmid onto the genome of *B. thetaiotaomicron*.<br>(Cell Syst. 2015; 1(1): 62-71) | atgaatatcaagcgcaacatcatttttgcattggagagccggaaaaagaac<br>ggtgtgccaatcgtagagaacgtacccatccgtatgcgtgtcatctttgccag<br>ccaacgcatcgagtttacaacgggctaccggattgacgtagccaaatggg<br>atgcagataagcagcgggtaaagagcggatgtaccaacaagctaaagc<br>aaagtgcagccgaaatcaatacggacttgctgaaatactatgccgaaatcc<br>agaatattttcaaggaatttgaggtgcaggaggtcatgccaacgacccaac<br>agttgaaggaagctttcaacatgagaatgaaagacaccagcgaagaaca<br>gccggaagaagcccctgtcagcttttgggaggtgttcgatgagtttgtaaaa<br>gagtgcggtaaccagaataactggacggcatccacctatgaaaaatttgca<br>gcagtgaggaaccacctcaaagagttcaaggaggatgcaacgttcaacta<br>tttcaacgagtttggattgaacgaatacgtcaacttcctgcgtgacaccaagg<br>atatgagaaacagcaccatcggcaagcaaatgggattcctcaaatggttcc<br>tgcgctggagcttcaagaaaggacatcatcagaacattgcatacgatacgtt<br>caaaccgaaactgaaaaccacctcgaaaaaagtaatcttcctgacttggg<br>atgaactgaacaagctgaaagactaccagatacccaaggataagcaata |

| | | |
|---|---|---|
| | | cctggaacgtgtgcgtgatgttttcctgttctgctgctttacgagtttgcggtattc ggatgttcgcaatctgaaaagaagcgatgtgaagtccgaccacatcgaaat aaccacagtcaagactgccgacagcctgacgattgaactgaacaaatac agcaaagccatactggacaaatacaaggacatccatttcgagaattacatg gctctgcccgtcatcagcaaccagaagatgaacgattacctgaaagagct gggcgaactggcagaaatcaacgagcctgtacgggaaacctactacaag ggaaatgaacgtattgatgaagtcacacccaaatacgctttgctcagtaccc atgcaggaagaaggacattcatctgcaatgcgctggctctcggaatcccgg cacaggtggtcatgaaatggacgggacacagcgactacaaagctatgaa accctacattgacatagcggatgatattaaggcaaatgccatgaacaagttt aatcaacta |
| *attN2* | Recombination site of pNBU2 plasmid with *attBT* sites on the genome of *B. thetaiotaomicron.* (Cell Syst. 2015; 1(1): 62-71) | cctgtctctccgcaaaaaacgct |
| TcR | Tetracycline resistance gene (Genbank: NZ_CYYO01000 017) | gtgcgtttcgacaatgcatctactgtagtatattattgcttaatccaaatgaatat tataaatttaggaattcttgctcacattgatgcaggaaaaacttccgtaaccga gaatctgctgtttgccagtggagcaacggaaaagtgcggccgtgtggataa tggtgacaccataacggactctatggatatagagaaacgtagaggaattac tgtccgggcttctacgacatctattatctggaatggagtgaaatgcaatatcatt gacactccgggacacatggattttattgcggaagtggagcggacattcaaa atgcttgatggagcagtcctcatcttatccgcaaaggaaggcatacaagcg cagacaaagttgctgttcagtactttacaaaagctgcaaatcccgacaattat atttatcaataagattgaccgtgccggtgtgaatttggagcgtttgtatatggat ataaaaacaaatctgtcgcaagatgtcctgtttatgcaaactgttgtcgatgg atcggtttatccggtttgctcccaaacatatataaaggaagaatacaaagaa tttgtatgcaaccatgacgacgatatattagaacgatatttggcggatagcga aatttcaccggctgattattggaatacgataatcgctcttgtggcaaaagcca aagtctatccggtgctacatggatcagcaatgttcaatatcggtatcaatgagt tgttggacgccatttcttcttttatacttcctccggcatcagtctcaaacagacttt cagcttatctctataagatagagcatgaccccaaagggcataaaagaagttt tcttaaaataattgacggaagtctgagacttcgagacgttgtaagaatcaac gattcggaaaaattcatcaagattaaaaatctaaagactatttatcagggca gagagataaatgttgatgaagtgggtgccaatgatatcgcgattgtagaag atatagaagattttcgaatcggagattatttaggtgctaaaccttgtttgattcaa ggattatctcatcagcatcccgctctcaaatcctccgtccggccaaataagcc cgaagagagaagcaaggtgatatccgctctgaatacattgtggattgaaga cccgtctttgtccttttccataaactcatatagtgatgaattggaaatctcgttata tggtttgacccaaaaggaaatcatacagacattgctggaagaacgattttcc gtaaaggtccattttgatgagatcaagactatctacaaagaacgacctataa aaaaggtcaataagattattcagatcgaagtaccacccaacccttactggg ccacaatagggctgactcttgaacccttaccgttaggggcaggggttgcaaat cgaaagtgacatctcctatggttatctgaaccattcttttcaaaatgccgttttg aagggattcgtatgtcttgccaatctggtttacatggatgggaagtgacagat ctgaaagtaacttttactcaagccgagtattatagcccggtaagtacacctgc tgatttcagacagctgaccccttatgtcttcaggctggctttgcaacagtcagg tgtggacattctcgaaccgatgctctgttttgagttgcagataccccaagtagc |

| | | |
|---|---|---|
| | | gagttccaaagctattacagatttgcaaaaactgatgtctgagattgaagac<br>atcagttgtaataatgagtggtgtcatattaaagggaaagttccattaaataca<br>agtaaagactatgcctcagaagtaagttcgtacactaagggcttaggcatttt<br>tatggttaagccatgtgggtatcaaataacaaaagacggttattctgataatat<br>ccgcatgaacgaaaaagataaacttttattcatgttccaaaaatcaatgtcatt<br>aaaataa |

**Supplementary Table 5. Plasmids used in experiments.**

| Name | Description | Purpose | Figure |
|---|---|---|---|
| pNH9104 | pNBU2-phes-$P_1$-NS-cas9-$P_{BT1311}$-rpiL*-nanoluc | First recombination | 2, S2 |
| pNH9040 | pNBU2-phes-$P_1$-NS-cas9-$P_{BT1311}$-crRCN-rpiL*-nanoluc | First recombination | 2, S2 |
| pNH9058 | pNBU2-phes-$P_1$-NS-cas9-$P_{BT1311}$-crR7N-rpiL*-nanoluc | First recombination | 2, S2 |
| pNH9059 | pNBU2-phes-$P_1$-NS-cas9-$P_{BT1311}$-crR10N-rpiL*-nanoluc | First recombination | 2, S2 |
| pNH9060 | pNBU2-phes-$P_1$-NS-cas9-$P_{BT1311}$-crR12N-rpiL*-nanoluc | First recombination | 2, S2 |
| pNH9106 | pNBU2-phes-$P_1$-taRNA1-cas9-$P_{BT1311}$-crRCN-rpiL*-nanoluc | First recombination | 2, S2, S3 |
| pNH9107 | pNBU2-phes-$P_1$-taRNA2-cas9-$P_{BT1311}$-crRCN-rpiL*-nanoluc | First recombination | 2, S2, S3 |
| pNH9108 | pNBU2-phes-$P_1$-taRNA7-cas9-$P_{BT1311}$-crRCN-rpiL*-nanoluc | First recombination | 2, S2, S3 |
| pNH9109 | pNBU2-phes-$P_1$-taRNA3-cas9-$P_{BT1311}$-crRCN-rpiL*-nanoluc | First recombination | 2, S2, S3 |
| pNH9142 | pNBU2-phes-$P_1$-taRNA4-cas9-$P_{BT1311}$-crRCN-rpiL*-nanoluc | First recombination | 2, S2, S3 |
| pNH9143 | pNBU2-phes-$P_1$-taRNA5-cas9-$P_{BT1311}$-crRCN-rpiL*-nanoluc | First recombination | 2, S2, S3 |
| pNH9144-2 | pNBU2-phes-$P_1$-taRNA6-cas9-$P_{BT1311}$-crRCN-rpiL*-nanoluc | First recombination | 2, S2, S3 |
| pNH9169 | pNBU2-phes-$P_{BT1311}$-NS-cas9-$P_1$-rpiL*-nanoluc | First recombination | 2, 4, S2 |
| pNH9168 | pNBU2-phes-$P_{BT1311}$-NS-cas9-$P_1$-crRCN-rpiL*-nanoluc | First recombination | 2, S2 |
| pNH9188 | pNBU2-phes-$P_{BT1311}$-taRNA6-cas9-$P_1$-crRCN-rpiL*-nanoluc | First recombination, Evaluation of HGT | 2-7, S1-S11 |
| pNH9195 | pNBU2-phes-$P_{cfxA}$-sgRNA1 | Second recombination | 3, S2 |
| pNH9196 | pNBU2-phes-$P_{cepA}$-sgRNA1 | Second recombination | 3, 4, 6, 7, S1, S2, S4, S5, S6, S8, S9, S10, S11 |
| pNH9198 | pNBU2-phes-$P_{cepA}$-NT | Second recombination | 4, 7 S2, S6, |

| | | | |
|---|---|---|---|
| | | | S10, S11 |
| pYL100 | pNBU2-NBU2 integrase-*attN*-$P_{cepA}$-sgRNA1-emR | Integration of a plasmid | 4, S7 |
| pYL114 | pNBU2-NBU2 integrase-*attN*-$P_{cepA}$-NT-emR | Integration of a plasmid | 4, S7 |
| pYL101 | pNBU2-NBU2 integrase-*attN*-$P_{cepA}$-sgRNA12-emR | Integration of a plasmid | 4, S7 |
| pYL103 | pNBU2-NBU2 integrase-*attN*-$P_{cepA}$-sgRNA13-emR | Integration of a plasmid | 4, S7 |
| pYL106 | pNBU2-NBU2 integrase-*attN*-$P_{cepA}$-sgRNA15-emR | Integration of a plasmid | 4, S7 |
| pYL107 | pNBU2-NBU2 integrase-*attN*-$P_{cepA}$-sgRNA16-emR | Integration of a plasmid | 4, S7 |
| pYL112 | pNBU2-NBU2 integrase-*attN*-$P_{cepA}$-sgRNA20-emR | Integration of a plasmid | 4, S7 |
| pNH9223 | pNBU2-phes-*thyA*-emR | Evaluation of HGT | 4, 6, 7 S6, S8, S11 |
| pNH9233 | pNBU2-phes-Mutated *thyA*-emR | Evaluation of HGT | 4, 6, S6 |
| pNH9225 | pNBU2-phes-$P_{BT1311}$-taRNA6-$P_{cfxA}$-sgRNA1-cas9-$P_1$-crRCN-rpiL*-nanoluc | Evaluation of HGT | 5 |
| pNH9226 | pNBU2-phes-$P_{BT1311}$-taRNA6-$P_{cepA}$-sgRNA1-cas9-$P_1$-crRCN-rpiL*-nanoluc | Evaluation of HGT | 5, 7 |
| pNH9227 | pNBU2-phes-$P_{BT1311}$-taRNA6-$P_{cepA}$-NT-cas9-$P_1$-crRCN-rpiL*-nanoluc | Evaluation of HGT | 5 |
| pNH9216 (Cell Syst. 2015; 1(1): 62-71) | pNBU2-NBU2 integrase-*attN*-emR | Integration of a plasmid | 7, S9, S10 |
| pNH9230-2 | pNBU2-NBU2 integrase-*attN*-tcR | Integration of a plasmid | 7, S9 |

**Supplementary Table 6. Primers used in experiments.**

| Name | Description | Figure | Sequence |
|---|---|---|---|
| Seq-*thyA*-F | PCR identification of horizontal gene transfer of the synthetic gene cassettes. | 5 | cactgtacgcagctaacac |
| mmD663 | PCR identification of horizontal gene transfer of the synthetic gene cassettes. | 5 | atcaccagcgtaccgtaatg |
| qPCR-EmR-F | PCR identification of horizontal gene transfer of the EmR gene. | 5, S8 | aacagcaatgctagtgatctg |
| qPCR-EmR-R | PCR identification of horizontal gene transfer of the EmR gene. | 5, S8 | atttggcagcataccttacaac |
| Rec-5'-*thyA*-F | PCR identification of integration of the synthetic gene cassettes. | S2 | attcattgatttattcagcgccatc |
| Rec-*thyA*-3'-R | PCR identification of integration of the synthetic gene cassettes and the sgRNA1. | S2 | agatgctttagatgagcaaattct g |
| CRI-sgRNA-3'-F | PCR identification of integration of the sgRNA1. | S2 | ggaacgaatggggttttagag |
| CRI-sgRNA5-3'-F | PCR identification of integration of the sgRNA (Non-targeting control). | S2 | gtggattctctagactttacttgag |
| *thyA*-F | PCR identification of horizontal gene transfer of the *thyA* gene. | S6 | tgttcggacatcagatgcgtttc |
| *thyA*-R | PCR identification of horizontal gene transfer of the *thyA* gene. | S6 | ggaagaaggcatgacaggga g |
