# Supplementary material for: Cas9-assisted biological containment of a genetically engineered human commensal bacterium and genetic elements
Source: Nat Commun. 2024 Mar 7;15:2096. doi: 10.1038/s41467-024-45893-w (PMC10920895; doi:10.1038/s41467-024-45893-w)
Supplement: Supplementary file 2 — Reporting Summary [file 41467_2024_45893_MOESM2_ESM.pdf]

nature portfolio

Corresponding author(s): Timothy K. Lu, Mark Mimee

Last updated by author(s): Jan. 31th. , 2024

Reporting Summary

Nature Portfolio wishes to improve the reproducibility of the work that we publish. This form provides structure for consistency and transparency in reporting. For further information on Nature Portfolio policies, see our Editorial Policies and the Editorial Policy Checklist.

Statistics

For all statistical analyses, confirm that the following items are present in the figure legend, table legend, main text, or Methods section.

n/a	Confirmed	
☐	☒	The exact sample size (*n*) for each experimental group/condition, given as a discrete number and unit of measurement
☐	☒	A statement on whether measurements were taken from distinct samples or whether the same sample was measured repeatedly
☐	☒	The statistical test(s) used AND whether they are one- or two-sided<br>*Only common tests should be described solely by name; describe more complex techniques in the Methods section.*
☒	☐	A description of all covariates tested
☐	☒	A description of any assumptions or corrections, such as tests of normality and adjustment for multiple comparisons
☐	☒	A full description of the statistical parameters including central tendency (e.g. means) or other basic estimates (e.g. regression coefficient) AND variation (e.g. standard deviation) or associated estimates of uncertainty (e.g. confidence intervals)
☐	☒	For null hypothesis testing, the test statistic (e.g. *F*, *t*, *r*) with confidence intervals, effect sizes, degrees of freedom and *P* value noted<br>*Give P values as exact values whenever suitable.*
☒	☐	For Bayesian analysis, information on the choice of priors and Markov chain Monte Carlo settings
☒	☐	For hierarchical and complex designs, identification of the appropriate level for tests and full reporting of outcomes
☒	☐	Estimates of effect sizes (e.g. Cohen's *d*, Pearson's *r*), indicating how they were calculated

Our web collection on statistics for biologists contains articles on many of the points above.

Software and code

Policy information about availability of computer code

Data collection	RNAfold WebServer and NUPACK web server were used to predict nucleic acid structures and their Minimum Free Energy.
Data analysis	Demultiplexing, quality control and adapter trimming of pair-end sequencing reads were performed with BCL Convert (Version 4.1.5). The sequencing reads were assembled by SPAdes (Version 3.15.2), and also mapped to reference sequences with Bowtie 2 (Version 2.4.5) for whole genome sequencing. GraphPad Prism 9 was used for statistic analysis.

For manuscripts utilizing custom algorithms or software that are central to the research but not yet described in published literature, software must be made available to editors and reviewers. We strongly encourage code deposition in a community repository (e.g. GitHub). See the Nature Portfolio guidelines for submitting code & software for further information.

Data

Policy information about availability of data

All manuscripts must include a data availability statement. This statement should provide the following information, where applicable:

- Accession codes, unique identifiers, or web links for publicly available datasets
- A description of any restrictions on data availability
- For clinical datasets or third party data, please ensure that the statement adheres to our policy

Data supporting this study are presented in the main text and Supplementary information, and source data are provided with this paper. Raw sequencing data and assembled genomes of whole-genome sequencing of Bacteroides thetaiotaomicron strains have been deposited at DDBJ/ENA/GenBank under the accession number: PRJNA754595. Plasmids used in this study are available from the corresponding author upon request.

Research involving human participants, their data, or biological material

Policy information about studies with human participants or human data. See also policy information about sex, gender (identity/presentation), and sexual orientation and race, ethnicity and racism.

Reporting on sex and gender	N/A
Reporting on race, ethnicity, or other socially relevant groupings	N/A
Population characteristics	N/A
Recruitment	N/A
Ethics oversight	N/A

Note that full information on the approval of the study protocol must also be provided in the manuscript.

Field-specific reporting

Please select the one below that is the best fit for your research. If you are not sure, read the appropriate sections before making your selection.

[x] Life sciences [ ] Behavioural & social sciences [ ] Ecological, evolutionary & environmental sciences

For a reference copy of the document with all sections, see nature.com/documents/nr-reporting-summary-flat.pdf

Life sciences study design

All studies must disclose on these points even when the disclosure is negative.

Sample size	Three or more biological replicates were performed for each experiment. No statistical method was used to predetermine sample size. We determined this sample size, considering allowable error size, need for statistical analysis and resources, based on previous studies by our group (Cell Syst 1, 62-71 (2015), Nat commun 10, 4028 (2019)) and articles in this field (Nature 548, 117-121 (2017), Nature Chemical Biology 12, 82-86 (2016)).
Data exclusions	No data were excluded.
Replication	All experiments other than the gnotobiotic animal experiment (Fig. 7d and e) , viability in various stress conditions (Supplementary Fig. 5) and in vivo genetic stability (Supplementary Fig. 11) were replicated at least twice and showed reproducible results. Three or four biological replicates demonstrated clear trends in the three experiments performed once. We determined one attempt is sufficient for them.
Randomization	Three or more random colonies of each strain were picked from agar plates for each experiment. Each strain was cultured and allocated to each experimental group by dividing the bacterial suspension. In the in vivo study, four mice were randomly allocated to each experimental group.
Blinding	No blinding was performed. Blinding is impossible as the investigators performed the experiments, allocating samples to each experimental group and being aware of name of bacterial strains, and controls and treated groups.

Reporting for specific materials, systems and methods

We require information from authors about some types of materials, experimental systems and methods used in many studies. Here, indicate whether each material, system or method listed is relevant to your study. If you are not sure if a list item applies to your research, read the appropriate section before selecting a response.

Materials & experimental systems

n/a	Involved in the study
☒	☐ Antibodies
☒	☐ Eukaryotic cell lines
☒	☐ Palaeontology and archaeology
☐	☒ Animals and other organisms
☒	☐ Clinical data
☒	☐ Dual use research of concern
☒	☐ Plants

Methods

n/a	Involved in the study
☒	☐ ChIP-seq
☒	☐ Flow cytometry
☒	☐ MRI-based neuroimaging

Animals and other research organisms

Policy information about studies involving animals; ARRIVE guidelines recommended for reporting animal research, and Sex and Gender in Research

Laboratory animals	Specific-pathogen-free female BALB/cJ mice (8 weeks) were purchased from The Jackson Laboratory, and four mice in each experimental group were housed together with a 12-hour light/ dark cycle at standard room temperature of 20-22 °C at a humidity of 30-70 %, and handled in non-sterile conditions. Mice were fed an irradiated mouse diet (ProLab IsoPro RMH3000, LabDiet). Female germ-free C57BL/6 mice were bred and housed in Trexler-style flexible film isolators (Class Biologically Clean) within polycarbonate mouse cages. Mice were weaned at 21 days of age and were fed an autoclaved, plant-based diet (JL Rat and Mouse/Auto 6F 5K67, LabDiet). All mice were housed in cages with a 12-hour light/ dark cycle at standard room temperature of 20-24 °C at a humidity of 30-70 %. 8-12 week-old mice were used for the experiment of in vivo bacterial conjugation.
Wild animals	This study did not involve any wild animal.
Reporting on sex	Female mice were used in this study as they tend to be less aggressive and be housed more socially.<br>In our study, mice were used to validate functionality of the Engineered Riboregulator, CRISPR Device and stability of thymidine auxotrophy of bacterial strains. No potential sex-specific effect is expected.
Field-collected samples	No field collected samples were used in the study
Ethics oversight	The protocols of specific-pathogen-free animal experiments were approved by the MIT Committee on Animal Care. The protocols of gnotobiotic animal experiments were performed in accordance with the Guide for the Care and Use of Laboratory Animals (8th ed.) and were approved by the Institutional Animal Care and Use Committee of the University of Chicago.

Note that full information on the approval of the study protocol must also be provided in the manuscript.

Plants

Seed stocks	N/A
Novel plant genotypes	N/A
Authentication	N/A
